# Case Report: Giant congenital hydronephrosis presenting as massive retroperitoneal cystic lesion with literature review

**DOI:** 10.3389/fsurg.2026.1763193

**Published:** 2026-09-29

**Authors:** Wen Yan, Mengmeng Xiao, Ming yi Li, Yin li Zhang, Ying chao Qin

**Affiliations:** 1 Department of Retroperitoneal Tumor Surgery, Peking University People's Hospital, Beijing, China; 2 Department of Medical Oncology, The Sixth Medical Center of Chinese PLA General Hospital, Beijing, China; 3 Department of Pathology, Peking University People's Hospital, Beijing, China; 4 Department of General Surgery, Shuangqiao Hospital, Beijing, China

**Keywords:** case report, giant congenital hydronephrosis, retroperitoneal cystic lesion, surgical resection, ureteropelvic junction obstruction

## Abstract

Giant retroperitoneal cystic lesions encompass multiple differential diagnoses including renal cysts, lymphangiomas and congenital hydronephrosis. Congenital giant hydronephrosis is extremely rare in adult patients, with limited clinical data to guide preoperative differentiation and surgical management. We report a unique case of a 60-year-old male with a 10-year history of progressive giant left retroperitoneal cystic mass, accompanied by left abdominal distending pain. Preoperative contrast-enhanced ultrasound and CT demonstrated severe left hydronephrosis with markedly atrophic renal cortex. Complete surgical resection of the cystic lesion was performed under laparotomy. Cyst fluid creatinine and postoperative histopathology confirmed the diagnosis of congenital giant hydronephrosis. The patient recovered uneventfully without complications during regular follow-up. We further review published literature to summarize clinical manifestations, multimodal imaging differentiation, surgical indications and long-term prognosis of adult giant congenital hydronephrosis, aiming to provide practical references for clinicians to distinguish retroperitoneal cystic masses and optimize surgical decision-making.

## 1 Background

Giant retroperitoneal cystic lesions include a variety of benign and pathological conditions, such as renal cysts, lymphangiomas, pancreatic pseudocysts, and congenital hydronephrosis. Accurate preoperative differentiation is crucial because these entities differ significantly in management and prognosis. Congenital hydronephrosis is usually associated with ureteropelvic junction obstruction and is more frequently encountered in pediatric patients (1, 2). In adults, giant congenital hydronephrosis presenting as a large retroperitoneal cystic mass is extremely rare and may be misdiagnosed preoperatively (3). To date, there is no widely accepted diagnostic algorithm or standardized surgical approach for this condition in adult patients (4). This case report describes a 60-year-old male with giant congenital hydronephrosis who presented as a retroperitoneal cystic lesion. The diagnosis was confirmed by cyst fluid creatinine level and postoperative histopathology. We summarize the diagnostic process, surgical treatment, and follow-up outcome, with a brief literature review focusing on the differential diagnosis and management of adult giant congenital hydronephrosis. Although abundant literature has described the clinical characteristics and surgical treatment of pediatric congenital hydronephrosis, adult patients presenting with giant congenital hydronephrosis forming a large retroperitoneal cystic mass are extremely scarce. At present, there is insufficient clinical evidence to distinguish this disease from other retroperitoneal cystic tumors before operation, and standardized diagnosis and treatment pathways have not been established. Therefore, this case report aims to fully elaborate the complete diagnostic process, surgical scheme and postoperative follow-up outcomes of this rare adult giant congenital hydronephrosis. Combined with a systematic literature review, we summarize the key differential imaging markers and optimal surgical intervention principles for similar giant retroperitoneal cystic lesions, to supplement clinical decision-making basis for frontline surgeons.

## 2 Case report

A 60-year-old male presented to our tertiary-level institution for evaluation of a giant retroperitoneal cystic lesion incidentally detected 10 years previously. Ten months before presentation, he developed progressive enlargement of the abdominal mass associated with left-sided abdominal distension and intermittent pain. There was no history of prior abdominal surgery; prior clinical assessment had raised a suspicion of “congenital absence of the left kidney". His medical history included over 10 years of hypertension, well-controlled with oral amlodipine 5 mg once daily and valsartan 80 mg once daily. Physical examination demonstrated visible left-sided abdominal bulging, with a large palpable soft-tender mass within the left abdominal quadrant. No peritoneal irritation signs were noted. Patient perspective: The patient expressed persistent anxiety regarding the long-standing mass, with concerns about malignant transformation and worsening symptoms. After detailed preoperative counselling, he consented to surgical resection. Postoperatively, abdominal distension and pain fully resolved, and the patient reported satisfactory recovery and improved quality of life at follow-up. The complete clinical timeline of this patient is summarized in Table 1.

**Table 1:** Timeline summarizing the patient's clinical course.

Time point	Clinical events
Past 10 years	Incidentally detected left retroperitoneal cystic lesion;remained asymptomatic
10 months before admission	Progressive left-sided abdominal distension and intermittent pain developed
Admission	Admitted to tertiary hospital for further evaluation and surgical treatment
Aug 8, 2025	Complete surgical resection of giant retroperitoneal cystic lesion
4-month postoperative follow-up	Complete relief of abdominal symptoms;no recurrence detected

Enhanced abdominal and pelvic CT revealed a giant retroperitoneal cystic structure on the left, multiloculated, maximum dimensions ∼30 × 20 × 34 cm, no enhancement; the lesion displaced and compressed surrounding structures, with thinning of the left renal cortex, highly suggestive of dilated left renal pelvis and calyces and possibly ureter. Right kidney size and morphology were acceptable, no abnormal densities or enhancement; liver size and morphology were normal (Figures 1A–C). A midline periumbilical incision was made. Intraoperatively, a giant multiloculated cyst was found in the left retroperitoneum, measuring ∼30 × 20 × 35 cm, soft, with a complete capsule, partially adherent tightly to the small intestine, separable by blunt and sharp dissection. The left renal vein was visible, the left renal cortex was thin and connected to the left renal vein, the inferior pole of the tumor revealed the ureter, which was identified at the inferior pole of the cyst; atresia was localized to the ureteropelvic junction, while the distal ureter remnant remained identifiable (Figure 2). The tumor was completely resected. Postoperative analysis of the cystic fluid reported: Creatinine 625 μmol/L (Figures 3A,B). Pathology report: (Retroperitoneum) Resection specimen: Submitted tissue consisted of multiloculated cystic structures, measuring ∼30 × 19 × 10 cm. The cyst walls contained atrophic glomeruli and renal tubule structures, partially composed of hyperplastic fibrous tissue with hyaline degeneration. Morphology consistent with multiloculated renal tissue, accompanied by renal tissue atrophy and sclerotic changes. (Figure 4A). Postoperative recovery was uneventful. The abdominal drain was removed on postoperative day 10. The patient achieved uneventful postoperative recovery and was discharged. He remained in regular outpatient follow-up 4 months after admission, with sustained resolution of abdominal symptoms.

**Figure 1:**
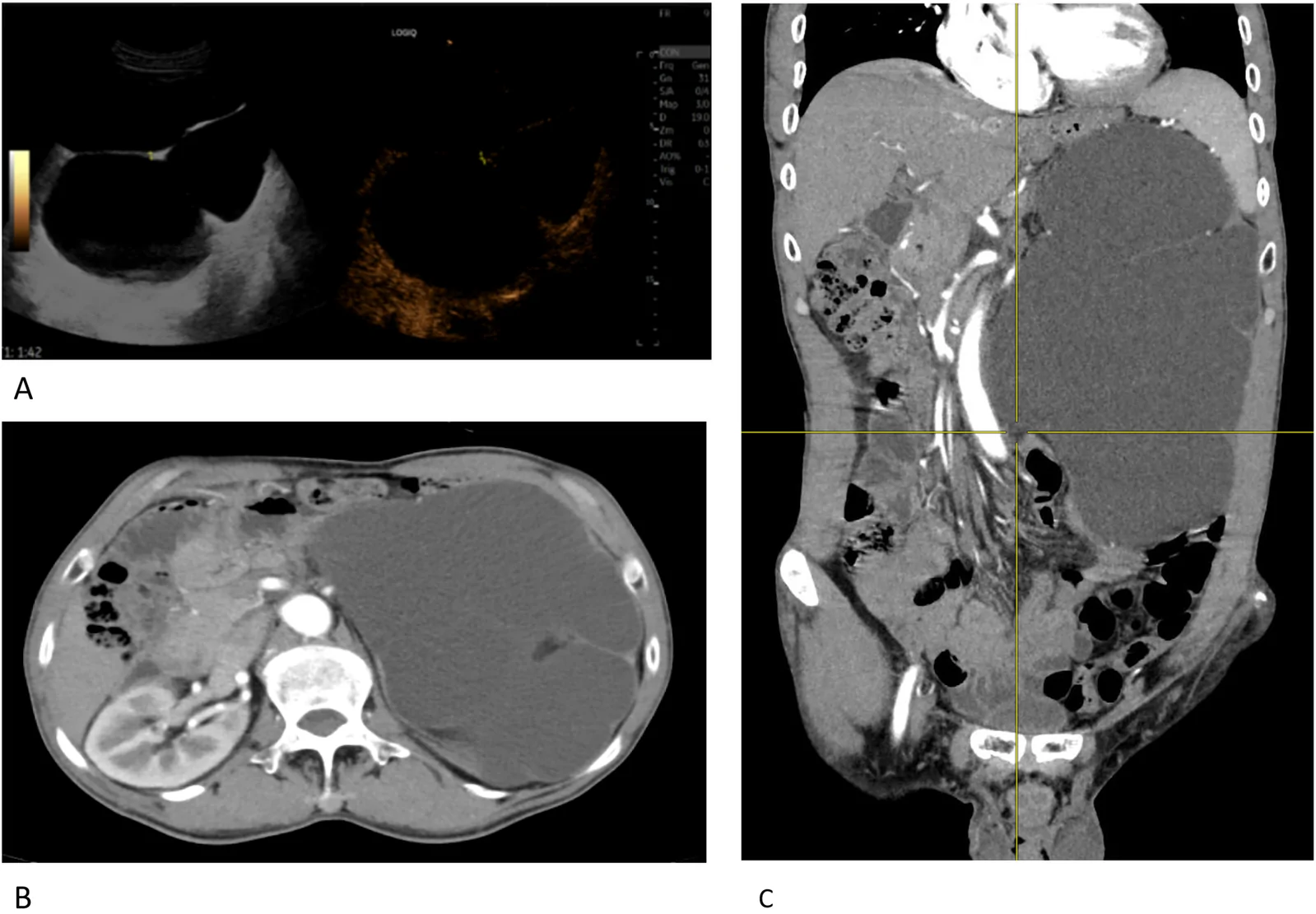
**(A)** Preoperative contrast-enhanced ultrasound of the abdomen: anechoic areas of varying sizes with internal septations are seen in the left renal region. **(B,C)** Preoperative computed tomography (CT): Reveals left-sided giant hydronephrosis with a significantly enlarged, fluid-filled collecting system and thinned renal parenchyma.

**Figure 2:**
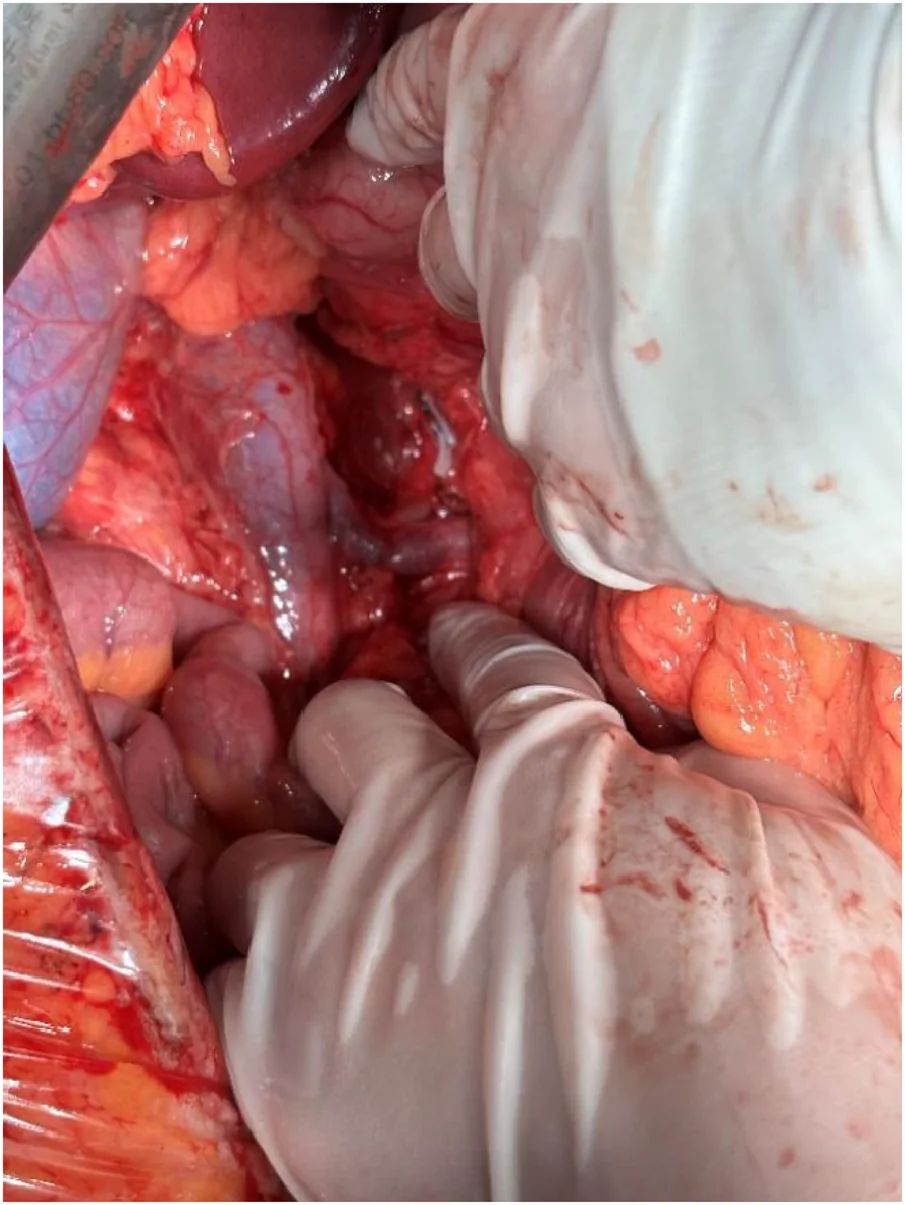
Intraoperative findings revealed that the giant cyst was connected to the inferior vena cava and abdominal aorta, corroborating the diagnosis of giant hydronephrosis leading to renal cortical atrophy.

**Figure 3:**
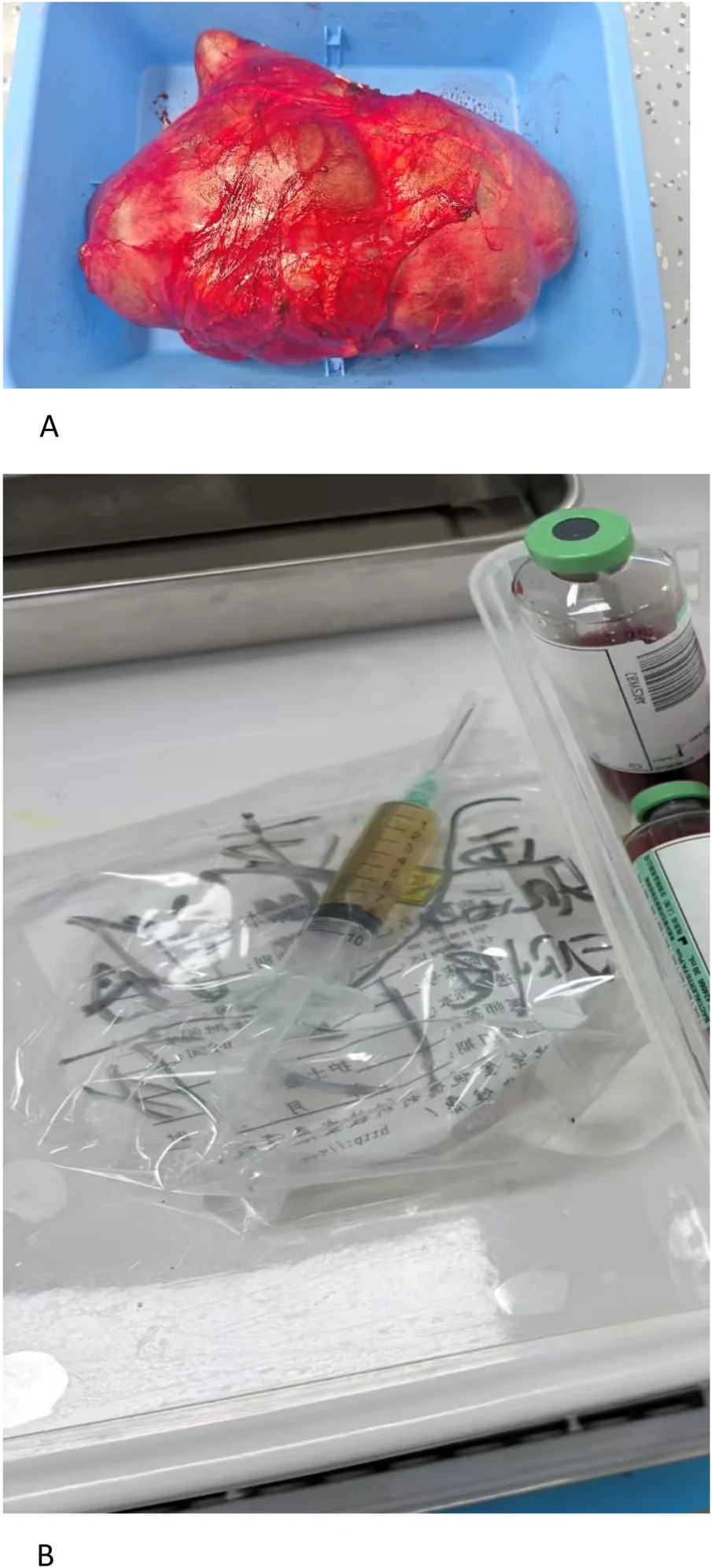
**(A)** postoperative resected specimen of giant hydronephrosis; **(B)** aspirated cyst fluid from the mass was submitted for laboratory analysis.

**Figure 4:**
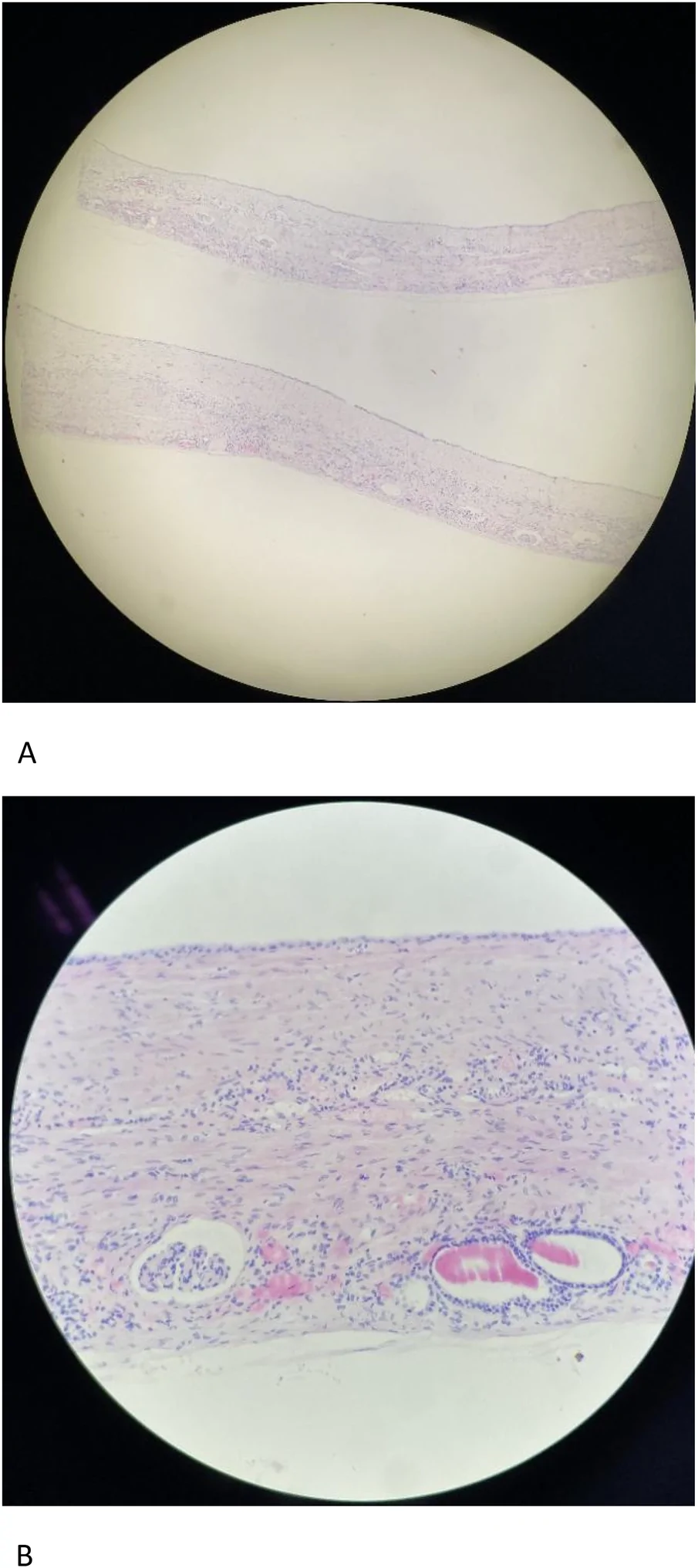
**(A)** histopathological analysis (H&E staining, 40x magnification) showed predominantly cystic structures lined by a single layer of cuboidal epithelium, with glomeruli and renal tubules observed within the cyst wall. **(B)** Histopathological analysis (H&E staining, 200x magnification) showed predominantly cystic structures lined by a single layer of cuboidal epithelium, with glomeruli and renal tubules observed within the cyst wall.

This retrospective single-case report was performed in accordance with the ethical principles of the Declaration of Helsinki. Written informed consent was obtained from the patient for publication of this case report, including clinical images, surgical records and pathological findings. All patient-identifiable information has been fully de-identified to protect patient privacy. Formal Institutional Review Board approval was waived for this single-patient case report, given that only anonymized routine clinical data were retrospectively reviewed, and no additional experimental interventions were performed.

## 3 Discussion

Giant retroperitoneal cystic lesions are relatively uncommon clinical entities in general surgical practice. Ovarian cystic masses are predominantly confined to the pelvic cavity, while cystic lymphangiomas and giant simple renal cysts demonstrate a clear female predominance in clinical statistics (5). Congenital giant hydronephrosis is a disorder traditionally identified and managed in pediatric populations (6); large, symptomatic lesions that first present during adulthood are extremely rare clinical scenarios, which makes our present case worthy of report and discussion.

Ureteropelvic junction obstruction (UPJO) is the dominant pathogenic factor of congenital giant hydronephrosis, covering two major categories: intrinsic ureteral luminal stenosis and extrinsic compression from aberrant lower-pole renal arteries. Beyond mechanical anatomical obstruction, two additional pathogenic mechanisms have been validated in existing research. The first is abnormal embryonic gene regulation that disrupts ureteral peristalsis and results in functional urinary outflow obstruction. The second is chronic sterile inflammation of the ureteral wall, which induces local tissue hyperplasia and luminal narrowing (7–10). After years of persistent obstruction, progressive ischemia and fibrosis will lead to diffuse thinning of renal cortex. Consistent with the clinical characteristics of our patient, most adults with this disease have no obvious pain, dysuria or hematuria in the early stage, and they only seek medical care when the cystic lesion expands into a palpable huge abdominal mass.

Long-term urinary tract obstruction triggers a cascade of renal parenchymal injuries, including oxidative stress, tubular epithelial cell apoptosis and progressive interstitial fibrosis. Multiple rodent model studies have confirmed that MMP-2 and TGF-*β*/ALK5 signaling pathways are core molecular mediators that accelerate renal fibrotic remodeling after obstruction (11–15). In clinical routine follow-up, traditional examinations such as renal function blood tests and urinary ultrasound cannot quantitatively reflect the severity of obstructive renal damage. Urinary molecular biomarkers have become a reliable non-invasive monitoring tool. Recognized biomarkers include TGF-β1, MCP-1, NGAL and KIM-1, and their urine concentrations are positively correlated with the degree of hydronephrosis. Emerging urinary proteomics research further screens novel differential proteins, which provides new ideas for early auxiliary diagnosis of obstructive nephropathy (16–19).

Clinically, giant congenital hydronephrosis is highly prone to preoperative misdiagnosis, and the two most easily confused diseases are simple renal cysts and multicystic dysplastic kidney (MCDK). A critical point to clarify is that the Bosniak classification system is only used for risk stratification of acquired renal cystic lesions in adults, and it cannot evaluate the developmental malformations of fetal renal tissues such as MCDK (20). These three cystic diseases share highly similar clinical manifestations: patients mostly only present with painless abdominal masses. If left untreated for a long time, all of them may cause compression of surrounding abdominal viscera, secondary intracystic infection, renal function decline or spontaneous cyst rupture (21, 22).

It is important to distinguish this entity from late-presenting multicystic dysplastic kidney (MCDK). Classic MCDK is characterized by complete full-length ureteral atresia, multiple non-communicating cystic compartments, and absence of functional glomerular-tubular renal parenchyma. In our case, preoperative CT demonstrated a single communicating dilated pelvicalyceal system rather than disconnected independent cysts. Intraoperatively, ureteral atresia was confined to the UPJ and the distal ureter remnant could still be identified. Histopathology confirmed residual atrophic glomeruli and renal tubules within the cyst wall. These combined imaging, intraoperative and histopathological findings support the diagnosis of giant hydronephrosis secondary to congenital UPJO, rather than MCDK.

Ultrasound is the preferred primary screening imaging modality for abdominal cystic masses, yet it lacks sufficient specificity to distinguish hydronephrosis from isolated renal cysts. Contrast-enhanced CT is the gold standard for preoperative differential diagnosis: giant hydronephrosis features diffuse thinning of renal cortex and multiple communicating cystic locules connected to the pelvicalyceal collecting system, while simple renal cysts show uniform thin walls without internal communicating compartments or septal enhancement (23). When imaging manifestations are still ambiguous and cannot confirm the connection between the cyst and urinary tract, intraoperative biochemical detection of cyst fluid creatinine and urea nitrogen can serve as a definitive diagnostic basis (24).

Retroperitoneal cystic lymphangioma is another essential differential diagnosis for large retroperitoneal cystic masses. It originates from congenital malformation of lymphatic drainage vessels, and complete surgical resection is the first-line treatment to reduce long-term recurrence risk (25–28). Contrast-enhanced ultrasound (CEUS) is an auxiliary imaging method with unique advantages. It does not involve ionizing radiation and can sensitively display microvascular perfusion inside cyst walls and septa, helping clinicians exclude cystic malignant tumors (29, 30).

Accurate preoperative differentiation of these retroperitoneal cystic lesions is of great clinical significance, because their surgical schemes and intraoperative management principles differ significantly. Misdiagnosis before surgery may lead to inappropriate operation plans and bring unnecessary trauma to patients. When surgeons encounter adult patients with huge retroperitoneal cystic masses, giant congenital hydronephrosis must be included in the differential diagnosis list. Combined application of multimodal imaging and intraoperative cyst fluid biochemical analysis can realize accurate diagnosis and formulate individualized optimal surgical strategies.

## Conclusion

This case illustrates the clinical features of late-diagnosed congenital hydronephrosis in adulthood, a rare clinical entity. Long-term asymptomatic progression may delay clinical recognition until massive cystic distension and pain occur.Contrast-enhanced CT serves as the core preoperative imaging modality. Intra-operative cyst-fluid biochemical assessment reliably verifies communication with the urinary collecting system. Surgeons should keep this disease in mind for large retroperitoneal cystic masses in adults to avoid sub-optimal surgical strategies.

## Data Availability

The original contributions presented in the study are included in the article/Supplementary Material, further inquiries can be directed to the corresponding author.

## References

[1] Nguyen HT Herndon CD Cooper C Gatti J Kirsch A Kokorowski P. The society for fetal urology consensus statement on the evaluation and management of antenatal hydronephrosis. J Pediatr Urol. (2010) 6(3):212–31. 10.1016/j.jpurol.2010.02.205 20399145

[2] Hamilton BE Martin JA Ventura SJ. Births: preliminary data for 2012. Natl Vital Stat Rep. (2013) 62(3):1–20. 24321416

[3] Joseph M Darlington D. Acute presentation of giant hydronephrosis in an adult. Cureus. (2020) 12(6):e8702. 10.7759/cureus.8702 32699698 PMC7370684

[4] Boşoteanu M Boşoteanu C Deacu M Aşchie M Bordei P. Etio-pathogenic and morphological correlations in congenital hydronephrosis. Rom J Morphol Embryol. (2011) 52(1):129–36. 21424044

[5] Mansour S Kluger Y Khuri S. Adult primary retroperitoneal lymphangioma: updated facts. World J Oncol. (2023) 14(1):15–20. 10.14740/wjon1561 36896002 PMC9990737

[6] Warady BA Chadha V. Chronic kidney disease in children: the global perspective. Pediatr Nephrol. (2007) 22(12):1999–2009. 10.1007/s00467-006-0410-1 17310363 PMC2064944

[7] Siddiqui MM McDougal WS. Urologic assessment of decreasing renal function. Med Clin North Am. (2011) 95(1):161–8. 10.1016/j.mcna.2010.08.031 21095419

[8] Isali I McClellan P Wong TR Gupta S Woo L. A systematic review of underlying genetic factors associated with ureteropelvic junction obstruction in stenotic human tissue. J Pediatr Urol. (2022) 18(5):629–41. 10.1016/j.jpurol.2022.07.022 35987676 PMC10152382

[9] Chen F. Genetic and developmental basis for urinary tract obstruction. Pediatr Nephrol. (2009) 24(9):1621–32. 10.1007/s00467-008-1072-y 19085015 PMC2844875

[10] Costigan CS Rosenblum ND. Understanding ureteropelvic junction obstruction: how far have we come? Front Urol. (2023) 3(1154740). 10.3389/fruro.2023.1154740 40778044 PMC12327334

[11] Ingraham SE Saha M Carpenter AR Robinson M Ismail I Singh S. Pathogenesis of renal injury in the megabladder mouse: a genetic model of congenital obstructive nephropathy. Pediatr Res. (2010) 68(6):500–7. 10.1203/PDR.0b013e3181f82f15 20736884 PMC3121911

[12] Forbes MS Thornhill BA Minor JJ Gordon KA Galarreta CI Chevalier RL. Fight-or-flight: murine unilateral ureteral obstruction causes extensive proximal tubular degeneration, collecting duct dilatation, and minimal fibrosis. Am J Physiol Renal Physiol. (2012) 303(1):F120–9. 10.1152/ajprenal.00110.2012 22535799 PMC3431140

[13] Forbes MS Thornhill BA Galarreta CI Minor JJ Gordon KA Chevalier RL. Chronic unilateral ureteral obstruction in the neonatal mouse delays maturation of both kidneys and leads to late formation of atubular glomeruli. Am J Physiol Renal Physiol. (2013) 305(12):F1736–46. 10.1152/ajprenal.00152.2013 24107422 PMC3882445

[14] Tveitarås MK Skogstrand T Leh S Helle F Iversen BM Chatziantoniou C. Matrix metalloproteinase-2 knockout and heterozygote mice are protected from hydronephrosis and kidney fibrosis after unilateral ureteral obstruction. PLoS One. (2015) 10(12):e0143390. 10.1371/journal.pone.0143390 PMC4687651 26673451

[15] Peleli M Al-Mashhadi A Yang T Larsson E Wåhlin N Jensen BL. Renal denervation attenuates NADPH oxidase-mediated oxidative stress and hypertension in rats with hydronephrosis. Am J Physiol Renal Physiol. (2016) 310(1):F43–56. 10.1152/ajprenal.00345.2015 26538440

[16] Merrikhi A Bahraminia E. Association of urinary transforming growth factor-*β*1 with the ureteropelvic junction obstruction. Adv Biomed Res. (2014) 3(123):123. 10.4103/2277-9175.133196 24949294 PMC4063104

[17] Taranta-Janusz K Wasilewska A Dębek W Waszkiewicz-Stojda M. Urinary cytokine profiles in unilateral congenital hydronephrosis. Pediatr Nephrol. (2012) 27(11):2107–13. 10.1007/s00467-012-2230-9 22744767 PMC3461208

[18] Wasilewska A Taranta-Janusz K Dębek W Zoch-Zwierz W Kuroczycka-Saniutycz E. KIM-1 and NGAL: new markers of obstructive nephropathy. Pediatr Nephrol. (2011) 26(4):579–86. 10.1007/s00467-011-1773-5 21279810 PMC3043232

[19] Ahram DF Lim TY Ke J Jin G Verbitsky M Bodria M. Rare single nucleotide and copy number variants and the etiology of congenital obstructive uropathy: implications for genetic diagnosis. J Am Soc Nephrol. (2023) 34(6):1105–19. 10.1681/ASN.0000000000000132 36995132 PMC10278788

[20] Gobishangar S Shelton J. Minimally invasive management of massive giant hydronephrosis in tertiary care centre northern Sri Lanka: a case report. Int J Surg Case Rep. (2021) 88(106514):106514. 10.1016/j.ijscr.2021.106514 34673471 PMC8528776

[21] Kaura KS Kumar M Sokhal AK Gupta AK Purkait B Saini D Sankhwar S. Giant Hydronephrosis: Still a Reality!. Turk J Urol. (2017) 43(3):337–44. 10.5152/tud.2017.78379 28861308 PMC5562255

[22] Yang WT Metreweli C. Giant hydronephrosis in adults: the great mimic. Early diagnosis with ultrasound. Postgrad Med J. (1995) 71(837):409–12. 10.1136/pgmj.71.837.409 7567732 PMC2397982

[23] Silverman SG Pedrosa I Ellis JH Hindman NM Schieda N Smith AD. Bosniak classification of cystic renal masses, version 2019: an update proposal and needs assessment. Radiology. (2019) 292(2):475–88. 10.1148/radiol.2019182646 31210616 PMC6677285

[24] Mountney J Chapple CR Johnson AG. Giant hydronephrosis–a diagnostic dilemma. Urol Int. (1998) 61(2):121–3. 10.1159/000030302 9873254

[25] Morais FP Carvalho IR Carvalho IR Luppi AM Dornelas BC. Child abdominal distension due to a massive retroperitoneal lymphangioma: a cyto-histopathological and clinical correlation. Cureus. (2024) 16(11):e72874. 10.7759/cureus.72874 39624567 PMC11610026

[26] Ferreira AM Reis RB Kajiwara PP Silva GE Jr EJ Muglia VF. MRI evaluation of complex renal cysts using the Bosniak classification: a comparison to CT. Abdom Radiol (NY. (2016) 41(10):2011–9. 10.1007/s00261-016-0797-5 27271286

[27] Suhani, Aggarwal L Ali S Thomas S. Giant retroperitoneal lymphangioma: a rare entity. Indian J Surg. (2014) 76(5):402–4. 10.1007/s12262-013-0989-y 26396474 PMC4571525

[28] DI Marco M Grassi E Vecchiarelli S Durante S Macchini M Biasco G. Retroperitoneal lymphangioma: a report of 2 cases and a review of the literature regarding the differential diagnoses of retroperitoneal cystic masses. Oncol Lett. (2016) 11(5):3161–6. 10.3892/ol.2016.4367 27123082 PMC4841079

[29] Mu J Mao Y Li F Xin X Zhang S. Superb microvascular imaging is a rational choice for accurate Bosniak classification of renal cystic masses. Br J Radiol. (2019) 92(1099):20181038. 10.1259/bjr.20181038 31067075 PMC6636258

[30] Shan K Fu ABDLAZZHL Liu N Cai Q Fu Q Liu L. Contrast-enhanced ultrasound (CEUS) vs contrast-enhanced computed tomography for multilocular cystic renal neoplasm of low malignant potential: a retrospective analysis for diagnostic performance study. Medicine (Baltimore. (2020) 99(46):e23110. 10.1097/MD.0000000000023110 33181678 PMC7668474

